# Nonfatal Firearm Injury and Firearm Mortality in High-risk Youths and Young Adults 25 Years After Detention

**DOI:** 10.1001/jamanetworkopen.2023.8902

**Published:** 2023-04-21

**Authors:** Nanzi Zheng, Karen M. Abram, Leah J. Welty, David A. Aaby, Nicholas S. Meyerson, Linda A. Teplin

**Affiliations:** 1 Department of Psychiatry and Behavioral Sciences, Northwestern University Feinberg School of Medicine, Chicago, Illinois; 2 Department of Preventive Medicine, Northwestern University Feinberg School of Medicine, Chicago, Illinois; 3 Department of Health Policy and Management, Johns Hopkins Bloomberg School of Public Health, Baltimore, Maryland

## Abstract

**Importance:**

Youths, especially Black and Hispanic males, are disproportionately affected by firearm violence. Yet, no epidemiologic studies have examined the incidence rates of nonfatal firearm injury and firearm mortality in those who may be at greatest risk—youths who have been involved with the juvenile justice system.

**Objectives:**

To examine nonfatal firearm injury and firearm mortality in youths involved with the juvenile justice system and to compare incidence rates of firearm mortality with the general population.

**Design, Setting, and Participants:**

The Northwestern Juvenile Project is a 25-year prospective longitudinal cohort study of 1829 youths after juvenile detention in Chicago, Illinois. Youths were randomly sampled by strata (sex, race and ethnicity, age, and legal status [juvenile or adult court]) at intake from the Cook County Juvenile Temporary Detention Center. Participants were interviewed at baseline (November 1995 to June 1998) and reinterviewed as many as 13 times over 16 years, through February 2015. Official records on mortality were collected through December 2020. Data analysis was conducted from November 2018 to August 2022.

**Main Outcomes and Measures:**

Participants self-reported nonfatal firearm injuries. Firearm deaths were identified from county and state records and collateral reports. Data on firearm deaths in the general population were obtained from the Illinois Department of Public Health. Population counts were obtained from the US census.

**Results:**

The baseline sample of 1829 participants included 1172 (64.1%) males and 657 (35.9%) females; 1005 (54.9%) Black, 524 (28.6%) Hispanic, 296 (16.2%) non-Hispanic White, and 4 (0.2%) from other racial and ethnic groups (mean [SD] age, 14.9 [1.4] years). Sixteen years after detention, more than one-quarter of Black (156 of 575 [27.1%]) and Hispanic (103 of 387 [26.6%]) males had been injured or killed by firearms. Males had 13.6 (95% CI, 8.6-21.6) times the rate of firearm injury or mortality than females. Twenty-five years after the study began, 88 participants (4.8%) had been killed by a firearm. Compared with the Cook County general population, most demographic groups in the sample had significantly higher rates of firearm mortality (eg, rate ratio for males, 2.8; 95% CI, 2.0-3.9; for females: 6.5; 95% CI, 3.0-14.1; for Black males, 2.5; 95% CI, 1.7-3.7; for Hispanic males, 9.6; 95% CI, 6.2-15.0; for non-Hispanic White males, 23.0; 95% CI, 11.7-45.5).

**Conclusions and Relevance:**

This is the first study to examine the incidence of nonfatal firearm injury and firearm mortality in youths who have been involved with the juvenile justice system. Reducing firearm injury and mortality in high-risk youths and young adults requires a multidisciplinary approach involving legal professionals, health care professionals, educators, street outreach workers, and public health researchers.

## Key Points

**Question:**

What are the incidence rates of nonfatal firearm injury and firearm mortality in youths who have been involved with the juvenile justice system?

**Findings:**

This 25-year longitudinal cohort study (n = 1829) found that youths involved with the juvenile justice system had up to 23 times the rate of firearm mortality as the general population; rates varied by sex, race and ethnicity, and age. Sixteen years after detention, more than one-quarter of Black and Hispanic males had been injured or killed by firearms.

**Meaning:**

These findings suggest that reducing firearm injury and mortality in high-risk youths and young adults requires a multidisciplinary approach involving legal professionals, health care professionals, educators, street outreach workers, and public health researchers.

## Teaser

This cohort study examines nonfatal firearm injury and firearm mortality in youths involved with the juvenile justice system in Chicago, Illinois, and compares incidence rates in this population with those in the general population.

## Introduction

The United States is facing a public health crisis as rates of firearm injury and death escalate. More than 100 deaths are caused by firearms each day.^1^ Youths and young adults—especially Black and Hispanic males—are disproportionately affected. In 2020, Black males ages 20 to 24 years had higher rates of overall firearm death and firearm homicide death than any other demographic group.^2^ Compared with non-Hispanic White males aged 20 to 24 years, Black males had more than 20 times and Hispanic males more than 3 times the rate of firearm homicide mortality.

Even more people are injured by firearms. In the United States, between 2009 and 2017, 234 people, on average, sustained nonfatal firearm injuries every day.^3^ In 2017, Black people were 10 times more likely than non-Hispanic White people to have been injured by firearms; Hispanic people were twice as likely to have been injured by firearms than non-Hispanic White people.^4^ Rates of nonfatal firearm injury are highest among young adult males in their early 20s.^5^

Many epidemiologic studies of firearm violence have investigated youths in the general population, studying those in the community (such as the Youth Risk Behavior Survey, the National Longitudinal Survey of Youth, and the National Crime Victimization Survey^6,7,8^) or using national databases (such as the Web-based Injury Statistics Query and Reporting System, the National Violent Death Reporting System, and the National Fatality Review Case Reporting System^9,10,11^). Other studies have sampled youths with firearm injuries seen in emergency departments or trauma centers.^12,13,14^

Far fewer epidemiologic studies have examined youths involved with the juvenile justice system. We reviewed the literature, searching PubMed, Google Scholar, PsychInfo, and Scopus for epidemiologic studies conducted in the United States of firearm injury or death that met the following criteria: (1) sampled youths who have been involved with the juvenile justice system; (2) published in a peer-reviewed journal since 1990; and (3) reported prevalence or incidence rates of firearm injury or death as outcome variables. We excluded studies that did not differentiate between deaths from firearms and those from other causes.^15,16^ Five studies met these criteria (eAppendix 1 in Supplement 1). None, however, reported incidence rates,^17,18,19,20,21^ a key limitation because only incidence rates allow for comparisons to youths in the general population.

Youths involved with the juvenile justice system are important to study because they are at great risk for firearm injury and death.^20,22,23^ These youths are exposed to numerous risk factors associated with firearm injury and death, including proximity to firearms^24^ and community violence,^23,25,26^ substance use,^21^ and gang activity.^20,22,27^

To our knowledge, this is the first study to examine the incidence rates of nonfatal firearm injury and firearm mortality in youths who have been involved with the juvenile justice system. Using data from the Northwestern Juvenile Project (NJP), we (1) examine sex and racial and ethnic differences in incidence rates of firearm injury and mortality and (2) compare incidence rates of firearm mortality in the NJP sample with those in the general population. Our data will help define the scope of the problem, guide the allocation of resources, and inform the development of preventive interventions.

## Methods

Additional details about methods appear in the eMethods in Supplement 1 and are published elsewhere.^20,21,24,28^ Participants signed either an assent form (<18 years) or a consent form (≥18 years). The institutional review boards of Northwestern University and the US Centers for Disease Control and Prevention approved all study procedures and waived parental consent for persons younger than 18 years, consistent with federal regulations regarding research with minimal risk.^29^ We nevertheless attempted to contact parents of minors to obtain their consent and to provide them with information on the study and used an independent participant advocate to represent the minors’ interests.^30^ This study followed the Strengthening the Reporting of Observational Studies in Epidemiology (STROBE) guidelines.^31^

### Samples and Procedures

We recruited a stratified random sample of 1829 youths who were arrested and detained at intake to the Cook County Juvenile Temporary Detention Center (CCJTDC) in Chicago, Illinois, between November 20, 1995, and June 14, 1998. CCJTDC is used for pretrial detention and for youths sentenced for fewer than 30 days. To ensure adequate representation of key subgroups, we stratified the NJP sample by sex, race, and ethnicity (Black, Hispanic, non-Hispanic White, and other racial and ethnic group, including Asian American and Pacific Islander and American Indian); age (10-13 years and ≥14 years); and legal status (processed in juvenile or adult court). The baseline sample included 1172 (64.1%) males and 657 (35.9%) females; 1005 (54.9%) Black, 524 (28.6%) Hispanic, 296 (16.2%) non-Hispanic White participants, and 4 participants (0.2%) from other racial and ethnic groups (mean [SD] age, 14.9 [1.4] years). We conducted face-to-face structured interviews at the detention center, most within 2 days of intake.

Follow-up interviews for the entire sample were conducted when funding became available, at approximately 3, 5, 6, 8, 12, 14, 15, and 16 years after the baseline interview; subsamples were interviewed at 3.5, 4, 10, 11, and 13 years after baseline. Participants were interviewed whether they lived in the community or in correctional facilities. At 16 years after baseline, we interviewed 1394 of 1709 participants who were still alive (81.6%).

### Variables

#### Data From the NJP

##### Nonfatal Firearm Injuries

At all follow-up interviews, we asked participants “since the last interview, have you had a serious physical injury you needed to take medicine for, or that required you to go to seek medical attention, or that limited your ability to do things?” If yes, we then asked, “what was it?”; “gunshot wound” was one of the response options.

##### Firearm Deaths

We obtained information on firearm deaths that occurred between November 1995 and December 2020 from: (1) death certificates from state medical examiners’ offices; (2) online news sources; (3) the National Death Index^32^; and (4) deaths reported by participants’ families, friends, and acquaintances. We were able to verify 84 of 88 firearm deaths (95.5%) with death certificates, online news sources, or the National Death Index.

#### General Population Data

We obtained counts of firearm deaths by sex (male, female), race and ethnicity (Black, Hispanic, non-Hispanic White), and age group (15-19, 20-24, 25-29, 30-24, 35-39 years) in Cook County from 2000, the earliest year available, to 2020 from the Illinois Department of Public Health. We obtained population counts from mid-year census estimates.^33,34^ No comparable data on nonfatal firearm injury are available for the general population (eAppendix 2 in Supplement 1).

### Statistical Analysis

We conducted all analyses using Stata version 15 (StataCorp) and its survey routines; we used StatTag to link Stata output to article text.^35^ To generate inferential statistics that reflect the population of CCJTDC, each participant was assigned a sampling weight to account for stratified sampling, augmented with nonresponse adjustments to account for missing data.^36,37^ We prespecified α = .05, and all tests were 2-tailed. We excluded from all models 4 participants who identified as other race and ethnicity.

#### The NJP Sample: Nonfatal Firearm Injury and Firearm Injury and Mortality Combined

We used Poisson regression with a log offset for time at risk to (1) generate the incidence rates and associated 95% CIs of nonfatal firearm injury and firearm injury and mortality and (2) compare rates of firearm injury and mortality by sex and race and ethnicity (self-reported). We estimated incidence rates separately for males and females. As in prior article,^21^ when estimating sex and racial and ethnic differences, we defined time at risk to include only days lived in the community. We excluded days incarcerated from time at risk because incarceration greatly reduces the risk of firearm injury and death; none of our participants were injured or killed by firearms while incarcerated.

#### Comparing the NJP Sample With the General Population: Firearm Mortality

We compared the incidence rates of firearm mortality and firearm homicide mortality between the NJP sample and the general population in Cook County by demographic groups. We used a period life table approach because firearm mortality rates change over time. For each age group, we compared the NJP sample with Cook County from the years in which our participants in that age group died. For example, because participants 20 to 24 years old died between 2000 to 2006, we compared them with Cook County residents aged 20 to 24 years between 2000 and 2006. We used Poisson regression to estimate incidence rates and associated SEs. Cook County rates were standardized to align with the sex and racial and ethnic distribution of the CCJTDC population. We used the delta method^38^ to calculate standard errors for rate ratios when comparing the incidence of mortality in the NJP sample with the Cook County general population. In this analysis, we could not adjust for time incarcerated because incarceration data are not available for the general population.

## Results

The baseline sample of 1829 participants included 1172 (64.1%) males and 657 (35.9%) females; 1005 (54.9%) Black, 524 (28.6%) Hispanic, 296 (16.2%) non-Hispanic White participants, and 4 participants (0.2%) from other racial and ethnic groups (mean [SD] age, 14.9 [1.4] years) (eTable in Supplement 1). By the 16-year follow-up interview, 302 participants (16.5%) had been injured or killed by a firearm; among Black and Hispanic males, more than one-quarter were injured or killed (Black, 156 of 575 [27.1%]; Hispanic, 103 of 387 [26.6%]). By December 2020, 25 years after the study began, 88 participants (4.8%) had been killed by a firearm (76 homicides, 7 suicides, 4 legal interventions [killed by police], 1 accidental death).

### Firearm Injury and Mortality in the NJP Sample

#### Sex and Racial and Ethnic Differences

For firearm injury and mortality, we observed the 1829 participants for a total of 25 039 person-years from baseline through the 16-year follow-up interviews. Table 1 reports the incidence rates of firearm injury and mortality (combined) by sex and by race and ethnicity within sex. Males were injured or killed by firearms at a rate of 2259 per 100 000 person-years (95% CI, 1871-2728 per 100 000 person-years); females, 235 per 100 000 person-years (95% CI, 155-356 per 100 000 person-years). Compared with females, males had 13.6 (95% CI, 8.6-21.6) times the rate of firearm injury or mortality.

**Table 1.** Incidence Rates of Combined Firearm Injury and Mortality Among the 1829 Participants in the Northwestern Juvenile Project

Characteristic	IR (95% CI)^a^	IRR (95% CI)^b^
**Full sample**		
Male	2259 (1871-2728)	13.6 (8.6-21.6)
Female	235 (155-356)	1 [Reference]
**Male** ^c^		
Black	2433 (1948-3038)	4.1 (2.5-6.8)
Hispanic	1961 (1513-2541)	3.2 (1.9-5.3)
Non-Hispanic White	756 (484-1179)	1 [Reference]
**Female** ^d^		
Black	244 (149-399)	1.0 (0.3-3.6)
Hispanic	193 (72-523)	0.8 (0.2-3.8)
Non-Hispanic White	241 (77-755)	1 [Reference]

Abbreviations: IR, incidence rate; IRR, incidence rate ratio.

^a^ Injury or death per 100 000 person-years.

^b^ IRRs are adjusted for time at risk in the community.

^c^ Black male vs Hispanic male: IRR, 1.3; 95% CI, 0.9-1.8.

^d^ Black female vs Hispanic female: IRR, 1.2; 95% CI 0.4-3.8.

Among males, there were significant racial and ethnic differences in firearm injury and mortality. Incidence rates were highest among Black males (2433 per 100 000 person-years; 95% CI, 1948-3038 per 100 000 person-years), followed by Hispanic males (1961; 95% CI, 1513-2541 per 100 000 person-years) and non-Hispanic White males (756; 95% CI, 484-1179 per 100 000 person-years). Compared with non-Hispanic White males, Black males had 4.1 (95% CI, 2.5-6.8) times and Hispanic males had 3.2 (95% CI, 1.9-5.3) times the rate of being injured or killed by firearm. There were no racial or ethnic differences among females.

Table 2 reports the incidence rates of nonfatal firearm injury by sex and by race and ethnicity within sex. Sex and racial and ethnic differences for nonfatal firearm injury were similar to those for firearm injury and mortality combined.

**Table 2.** Incidence Rates of Nonfatal Firearm Injury Among the 1829 Participants in the Northwestern Juvenile Project

Characteristic	IR (95% CI)^a^	IRR (95% CI)^b^
**Full sample**		
Male	1725 (1393-2136)	14.3 (8.4-24.2)
Female	174 (108-282)	1 [Reference]
**Male** ^c^		
Black	1855 (1442-2387)	4.6 (2.5-8.3)
Hispanic	1521 (1144-2022)	3.6 (1.9-6.5)
Non-Hispanic White	526 (309-895)	1 [Reference]
**Female** ^d^		
Black	183 (104-323)	0.8 (0.2-2.8)
Hispanic	97 (24-394)	0.4 (0.1-2.6)
Non-Hispanic White	241 (77-754)	1 [Reference]

Abbreviations: IR, incidence rate; IRR, incidence rate ratio.

^a^ Injury per 100 000 person-years.

^b^ IRRs are adjusted for time at risk in the community.

^c^ Black male vs Hispanic male: IRR, 1.3; 95% CI, 0.9-1.9.

^d^ Black female vs Hispanic female: IRR, 1.9; 95% CI, 0.4-8.4.

### Firearm Mortality: Comparison With the General Population

For firearm mortality, we observed the 1829 participants for a total of 40 504 person-years from baseline through December 2020. We compared incidence rates of firearm mortality (Figure) and firearm homicide mortality (eFigure in Supplement 1) in the NJP sample with those in the general population by demographic group.

**Figure.**
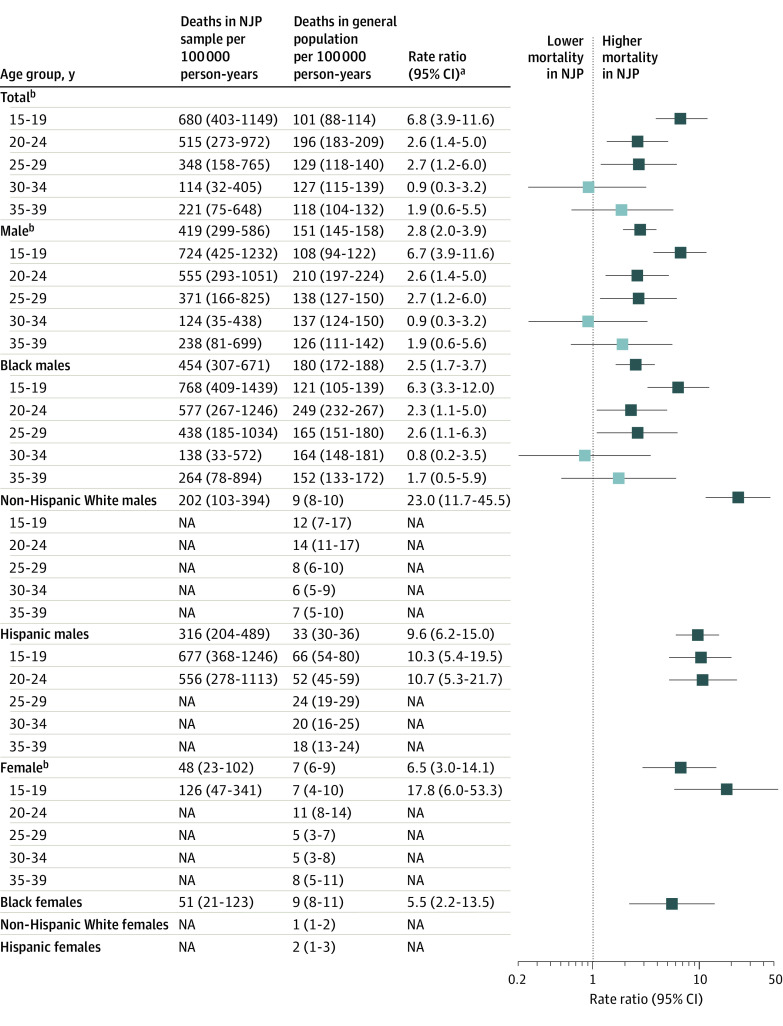
Standardized Firearm Mortality Rates in the Northwestern Juvenile Project (NJP) Compared With the General Population Not available (NA) indicates that there were 3 or fewer deaths in the NJP sample. Dark blue boxes indicate statistically significant difference in mortality rates (*P* < .05). ^a^Standard errors and 95% CIs estimated via the delta method. ^b^Rates across racial and ethnic categories are weighted to reflect the racial and ethnic distribution of the Cook County Juvenile Temporary Detention Center.

#### Males

Regardless of racial and ethnic group, males in the NJP sample had greater rates of firearm mortality compared with those in the general population, up through age 29 years. The magnitude of the difference, however, depended on age group and race and ethnicity. Black males ages 15 to 19 years in the NJP sample died by firearm at 6.3 (95% CI, 3.3-12.0) times the rate of those in the general population. Black males in their 20s had more than twice the rate of firearm mortality than those in the general population (20-24 years: rate ratio [RR], 2.3; 95% CI, 1.1-5.0; 25-29 years: RR, 2.6; 95% CI, 1.1-6.3). There were no significant differences in firearm mortality rates between the NJP sample and the general population for Black males ages 30 to 34 or 35 to 39 years. Hispanic males in the NJP sample had more than 10 times the rate of firearm mortality than those in the general population at ages 15 to 19 (RR, 10.3; 95% CI, 5.4-19.5) and 20 to 24 years (RR, 10.7; 95% CI, 5.3-21.7). Non-Hispanic White males in the NJP sample had more than 20 times the rate of firearm mortality than those in the general population (RR, 23.0; 95% CI, 11.7-45.5). There were too few firearm deaths among Hispanic males 25 years and older and non-Hispanic White males to estimate age-specific rates.

#### Females

Females in the NJP sample had more than 6 times the rate of firearm mortality than females in the general population (RR, 6.5; 95% CI, 3.0-14.1). Females aged 15 to 19 years had 17.8 times (95% CI, 6.0-53.3) the rate of firearm mortality than those in the general population. Overall, Black females had more than 5 times the rate of firearm mortality than those in the general population (RR, 5.5; 95% CI, 2.2-13.5). There were too few deaths among females to estimate specific rates for non-Hispanic White and Hispanic females or by age group.

## Discussion

To our knowledge, this is the first study to examine the incidence of nonfatal firearm injury and firearm mortality in youths who have been involved with the juvenile justice system. More than one-quarter of Black and Hispanic males in the NJP sample had been injured or killed by a firearm during 16 years of follow-up after detention. Sex and racial and ethnic differences mirror trends in the US general population.^2,4^ For example, males were injured or killed by firearms at 13 times the rate of females; Black and Hispanic males were injured or killed at significantly higher rates than non-Hispanic White males.

In most demographic subgroups, rates of firearm mortality were significantly higher in the NJP sample than in the general population. Young Hispanic males in the juvenile justice system had firearm mortality rates that are approximately 10 times higher than those for young Hispanic males in the general population; for non-Hispanic White males, 20 times higher. For Black males, the incidence rates of firearm mortality in the general population are so high^2^ that the magnitude of difference was smaller.

Why are rates of firearm injury and mortality so high in our juvenile justice sample? During adolescence, half of females and nearly three-quarters of males reported having easy access to firearms; one-quarter of males and 1 in 8 females were members of gangs that carried firearms.^24^ Access to firearms increases the risk for firearm homicide and suicide.^39^ Moreover, more than 70% of the NJP sample had experienced violence in the past^25^ (eg, witnessed violence, threatened with a weapon, injured by a firearm), which increases the risk of subsequent firearm-related violence, including threats, injuries, and mortality (referred to as *victimization* in the literature).^40^ These risk factors may also explain the disproportionately high rates of firearm injury and mortality in Black and Hispanic males, who were more likely to be involved with gangs and had access to firearms.^20,24,41^

### Implications for Public Policy

The juvenile justice system and schools should adopt trauma-informed practices. One of every 4 youths who survive a firearm injury are diagnosed with a mental health disorder within 1 year of the injury, most commonly trauma-related disorders, substance use disorders, and disruptive behavior disorders.^42^ Even indirect exposures to firearm violence—ie, witnessing an event or hearing gunshots—are associated with mental health problems.^43,44^ Mental health problems and concerns about safety may increase truancy, which may result in suspension or expulsion. Black and Hispanic children are the most vulnerable: they are more likely than non-Hispanic White children to be arrested at school for disruptive behaviors—behaviors that are often manifestations of trauma.^45^ Both juvenile justice facilities and schools must adopt trauma-informed practices to better address youths’ needs and to disrupt the school-to-prison pipeline,^46^ which disproportionately impacts racial and ethnic minority children. Trauma-informed practices (1) ensure that personnel learn how trauma affects youths’ behavior, (2) assess youths for trauma and mental health needs, and (3) connect youths who have experienced violence to mental health services.^47,48^

Medical centers should expand interventions for those who have experienced firearm violence. Youths and young adults who have been injured by firearms are at higher risk of subsequent firearm injury and mortality^12^ and of perpetrating firearm violence.^24^ Yet, few hospitals provide preventive interventions to reduce the likelihood of subsequent firearm-related violence^49,50,51^ and reduce aggressive behaviors.^49,52^ Interventions include assistance with conflict resolution^49,50,52^ and access to case management.^50,51^ Because hospital stays tend to be brief, intervention programs must connect patients to services in the community after discharge, including mental health services,^52,53^ substance abuse rehabilitation,^49^ education services,^49,51^ and employment training.^49^ Partnerships between hospital-based and community-based programs will better serve high-risk youths, interrupt the cycle of violence, and lower the risk of subsequent violence.^54^

### Implications for Future Research

A comprehensive national database of nonfatal firearm injuries should be established. The incidence of nonfatal injury is far higher than the incidence of firearm mortality. Yet, we could not compare the incidence of nonfatal firearm injuries in our sample with the general population because the available databases of nonfatal firearm injuries, eg, the National Electronic Injury Surveillance System (NEISS), the Nationwide Emergency Department Sample (NEDS), and the National Inpatient Sample (NIS), have limitations.^55^ First, these databases do not provide incidence rates by race and ethnicity,^3,56^ which limits the investigation of disparities. Second, these databases do not provide state- or county-level data, hampering geographic comparisons. Although some counties and states maintain their own databases, the type and quality of data varies considerably. Third, even the most comprehensive databases have limited generalizability. The NEDS and NEISS include records only from selected emergency departments. Similarly, the NIS collects records from a subset of hospitals, and they include only inpatient records,^57^ which would underestimate less serious injuries. Moreover, individuals with firearm injuries may not seek inpatient treatment in hopes of reducing the likelihood of police investigation.^58,59^ To address disparities in firearm injury in the United States, national databases must include data for nonfatal firearm injuries in emergency departments and inpatient and outpatient settings, collect accurate information on race and ethnicity, and provide state- and county-level data.

Future studies should investigate the cycle of being threatened with or injured by a firearm and the perpetration of violence. One study^24^ found that being injured by firearms in adolescence increases the risk of perpetrating firearm violence in adulthood. In turn, perpetrating firearm violence increases the risk of being threatened with or injured by a firearm.^40,60^ Our findings on firearm injury and mortality indicate that more research is needed. Longitudinal studies must examine (1) the sequence of being threatened with or injured by a firearm and perpetrating firearm violence as youths age and (2) the risk and protective factors for being threatened with or injured by a firearm and perpetrating firearm violence. Findings would identify where and how to interrupt this cycle.

### Limitations

This study has limitations. We may have underestimated the incidence rates of nonfatal firearm injury in our sample for three reasons: (1) we investigated only injuries that occurred after youths enrolled in the study; (2) we could not study the number of injuries sustained per person; and (3) we counted only injuries that required medical attention.

The difference in firearm mortality rates between youths in the NJP sample vs the general population might be larger than estimated because the populations are not mutually exclusive; youths involved with the juvenile justice system are also included in general population estimates. It was not feasible to study more than 1 jurisdiction. Our findings are generalizable only to other large urban areas with similar rates of firearm injury and mortality. Findings might not pertain to today’s detained youths. We could not adjust for socioeconomic status because there was little variability: nearly all study participants had economic disadvantage.

## Conclusions

In 2020, 39% more children and youths presented to hospitals with firearm injuries than in prior years.^61^ Firearms became the leading cause of death in children and youths for the first time since 1999; prior to 2020, automobile crash was the leading cause of death in children and youths.^62^ Reducing firearm injury and death in high-risk youths and young adults requires a multidisciplinary approach. Legal professionals, health care professionals, educators, street outreach workers, and public health researchers must collaborate to advocate for resources for those who experienced firearm violence, improve data collection on firearm injury and death, and facilitate hospital-, school-, and community-based preventive interventions.

## Notes

Supplement 1. **eMethods.** Expanded Notes on Study Methods **eTable.** Unweighted Sample Characteristics at Baseline **eFigure.** Standardized Firearm Mortality Rates in the Northwestern Juvenile Project (NJP) Compared With the General Population **eAppendix 1.** Literature Table: Studies of Firearm Injury or Mortality in Youths Involved With the Juvenile Justice System Since 1990, United States **eAppendix 2.** Summary of Nonfatal Firearm Injury Data in the General Population
eReferences.

Supplement 2.
Data Sharing Statement
