# Supplementary material for: Nonfatal Firearm Injury and Firearm Mortality in High-risk Youths and Young Adults 25 Years After Detention
Source: JAMA Netw Open. 2023 Apr 21;6(4):e238902. doi: 10.1001/jamanetworkopen.2023.8902 (PMC10122168; doi:10.1001/jamanetworkopen.2023.8902)
Supplement: Supplement 1. — eMethods. Expanded Notes on Study Methods eTable. Unweighted Sample Characteristics at Baseline eFigure. Standardized Firearm Mortality Rates in the Northwestern Juvenile Project (NJP) Compared With the General Population eAppendix 1. Literature Table: Studies of Firearm Injury or Mortality in Youths Involved With the Juvenile Justice System Since 1990, United States eAppendix 2. Summary of Nonfatal Firearm Injury Data in the General Population eReferences. [file jamanetwopen-e238902-s001.pdf]

Supplemental Online Content

Zheng N, Abram KM, Welty LJ, Aaby DA, Meyerson NS, Teplin LA. Nonfatal firearm injury and firearm mortality in high-risk youths and young adults 25 years after detention. *JAMA Netw Open.* 2023;6(4):e238902. doi:10.1001/jamanetworkopen.2023.8902

eMethods. Expanded Notes on Study Methods

eTable. Unweighted Sample Characteristics at Baseline

eFigure. Standardized Firearm Mortality Rates in the Northwestern Juvenile Project (NJP) Compared With the General Population

eAppendix 1. Literature Table: Studies of Firearm Injury or Mortality in Youth Involved With the Juvenile Justice System Since 1990, United States

eAppendix 2. Summary of Nonfatal Firearm Injury Data in the General Population

eReferences.

This supplemental material has been provided by the authors to give readers additional information about their work.

© 2023 Zheng N et al. *JAMA Network Open.*

eMethods. Expanded Notes on Study Methods

BACKGROUND

Characteristics of the Cook County Juvenile Temporary Detention Center (CCJTDC)

No single site can represent the entire country because jurisdictions may have different options for diversion and different demographics. We chose the detention center in Cook County (which includes Chicago and surrounding suburbs) for three reasons: First, nationwide, most detained youth live in and are detained in urban areas.[1] Second, Cook County has the third largest Hispanic population in the United States.[2] Studying Hispanic youth is important because they are overrepresented in the justice systems.[3] Finally, the detention center's size (daily census of approximately 650 youth and intake of 20 youth per day) insured that enough participants would be available.

Consistent with detained youth nationwide,[4] nearly 90% of detainees at CCJTDC were male; most were Black and Hispanic youth. CCJTDC's population was 77.9% Black, 5.6% non-Hispanic White, 16.0% Hispanic, and 0.5% other racial or ethnic groups.[3] The age and offense distributions of CCJTDC detainees are also similar to detained youth nationwide.

Sampling and Procedures

Northwestern Juvenile Project (*NJP*) participants include 1829 male and female youth, 10 to 18 years old, randomly sampled from intake into the CCJTDC from November 20, 1995, through June 14, 1998. The sampling was stratified by sex, race and ethnicity (Black, non-Hispanic White, Hispanic, and "other" race and ethnicity), age (10-13 years or ≥14 years), and legal status (processed in juvenile or adult court) to obtain enough participants to examine key subgroups (e.g., females, Hispanics, younger persons). There were 13 strata, as listed below. There were too few female detainees of each race and ethnicity and detainees identified as "other" race and ethnicity to further stratify these groups. Detainees aged 10 to 13 years were not stratified by legal status because they were generally too young to be considered for transfer to adult court.

Strata:

- Black females
- Non-Hispanic White females
- Hispanic females
- Black males, aged 10-13 years
- Non-Hispanic White males, aged 10-13 years
- Hispanic males, aged 10-13 years
- Black males, 14 years or older and processed as adult transfer
- Non-Hispanic White males, 14 years or older and processed as adult transfer
- Hispanic males, 14 years or older and processed as adult transfer
- Black males, 14 years or older and processed as a juvenile
- Non-Hispanic White males, 14 years or older and processed as a juvenile

© 2023 Zheng N et al. *JAMA Network Open.*

Hispanic White males, 14 years or older and processed as a juvenile
Other race and ethnicity

A study liaison was scheduled to work every day (including weekends) throughout the study. Each day, the liaison randomly selected potential participants within strata. Detainees were classified in strata using information listed in the intake log. The liaison sampled from the strata in a preset order. If no participants were available for a stratum, the liaison sampled from the next stratum. If multiple detainees were available for a stratum, the liaison used a random number table and the last digit of the CCJTDC ID number to randomly sample potential participants from within the stratum.[5] The final sampling fractions for the strata ranged from 0.018 to 0.689.

All detainees who were awaiting the adjudication or disposition of their case were eligible to participate in the study. Among these, 2275 detainees were randomly selected; 4.2% (34 youth and 62 parents or guardians) refused to participate. There were no significant differences in refusal by sex, race and ethnicity, or age. Twenty-seven youth left the detention center before an interview could be scheduled; 312 left CCJTDC while we attempted to locate their caretakers for consent. Eleven others were excluded from the sample because they were unable to complete the interview. The final sample size was 1829 participants: 1172 male, 657 female; 1005 Black, 296 non-Hispanic White, 524 Hispanic, 4 "other" race and ethnicity; age range, 10 to 18 years (mean, 14.9 years; median, 15 years) (see e**Table 1**).

The most common offenses were violent crimes (55.4%), property crimes (43.5%), violation of probation or parole (36.4%), drug crimes (30.7%), and weapons crimes (13.5%). (Numbers sum to >100% because participants could be arrested for more than 1 charge.)

Face-to-face structured interviews were conducted at the detention center in a private area, most within 2 days of intake. Participants were paid $25 for the 2- to 3-hour baseline interview.

We re-interviewed participants irrespective of where they lived: in the community (65%-69% of interviews); at correctional facilities (15%-29% of interviews); by telephone if they lived more than 2 hours away (4%-16% of interviews); or in a placement, such as a group home (<2% of interviews.) Participants were paid $50 for the 3- through 6-year follow-up interviews; $75 for the 8- through 13-year follow-up interviews; and $100 for the 14- through 16-year follow-up interviews, each lasting approximately 3 to 4 hours.

Youth Processed in Juvenile or Adult Court

Although most juvenile offenders are processed in juvenile court, all 50 states and the District of Columbia have legal mechanisms to try juveniles as adults in criminal court.[6,7] Transfers to adult criminal court typically result from: (1) judicial waiver on a case-by-case basis,[8-10] (2) automatic transfers based on the type of offense, criminal history, and age of the detainee,[8] and (3) prosecutorial direct-file mechanisms that allow prosecutors to determine when to file certain juvenile cases directly in adult criminal court.[8] The increased availability of legal mechanisms to process juveniles in adult criminal court is largely responsible for the 366% increase between 1983 and 1998 in the number of juveniles held in adult jails.[11] As of 2004, about 7% of the

approximately 2 million arrests of youths eligible for processing in the juvenile justice system were cases in which the youth was transferred directly to adult criminal court.[4,12]

Procedures for Obtaining Parental Consent for Minor Youth

For all interviews, participants signed either an assent form (if they were <18 years) or a consent form (if they were ≥18 years). The Northwestern University Institutional Review Board and the Centers for Disease Control and Prevention Institutional Review Board approved all study procedures and waived parental consent for persons younger than 18 years, consistent with federal regulations regarding research with minimal risk.[13] We nevertheless attempted to contact parents of minors to obtain their consent and to provide them with information on the study and used an independent participant advocate to represent the minors' interests.[13]

Baseline

Study liaisons tried to reach detainees' parents or guardians in 2 ways: first, they attempted to call them by telephone at least 3 times over 2 days. Second, they tried to obtain consent from the parents or guardians in person during visiting hours. A participant advocate acted on the child's behalf if the parents or guardians were not reachable. In the absence of a parent or guardian, the participant advocate protects the interests of the youth and determines that they are consenting voluntarily, understand the research procedure, and are not being coerced to participate. Consistent with federal regulations, we excluded detainees who did not wish to participate, even if their parents or guardians consented.[13,14]

Follow-up

Two weeks before a follow-up interview was due, a liaison telephoned the parent or guardian of minors to obtain their consent. If they provided consent, the liaison then contacted the youth to obtain assent and schedule their interview. The Illinois Department of Child and Family Services allowed us to recontact and interview participants who were under their guardianship, provided that we received assent from the youth. As with baseline interviews, we excluded detainees who did not wish to participate, even if their parents or guardians consented. Also as with baseline interviews, minors could still participate even if we could not reach their parent or guardian. If we could not reach them after 1 week and at least 5 attempts, we initiated the participant advocate system described above. In these cases, the liaison contacted the participant directly to request his or her assent. If we could not reach the participant by phone, an interviewer traveled to his or her location.[13,14]

Clinical Research Interviewers

For baseline and follow-up interviews, female participants were interviewed by female interviewers. Most interviewers had graduate degrees in psychology or an associated field and had experience interviewing at-risk youth; one-third were fluent in Spanish. All interviewers were trained for at least 1 month. Follow-up interviews were longer than baseline interviews because, at the request of our funding agencies, we added additional variables.

LITERATURE SEARCH

Methods

We searched PubMed, Google Scholar, PsychInfo, and Scopus for epidemiologic studies of firearm injury or death conducted in the United States that met the following criteria: (1) sampled youth who had been involved with the juvenile justice system; (2) published in a peer-reviewed journal since 1990; and (3) reported prevalence or incidence rates of firearm injury or mortality as outcome variables. Search terms included “firearm” OR “gun”, AND “death” OR “mortality” OR “injury”, AND “high-risk” OR “at-risk” OR “juvenile justice” OR “detained” OR “incarcerated”, AND “youth”. We excluded studies that did not differentiate between deaths from firearms and those from other causes.

MEASURES AND VARIABLES

Data on Nonfatal Firearm Injury in the *NJP* Sample

At all follow-up interviews, we asked participants “since the last interview, have you had a serious physical injury you needed to take medicine for, or that required you to go to seek medical attention, or that limited your ability to do things?” If yes, we then asked “what was it?”; “gunshot wound” was one of the response options. We did not collect data on the number of firearm injuries.

Data for Firearm Mortality in Cook County

We obtained counts of firearm deaths among Cook County residents between 2000 and 2020 from the Illinois Department of Public Health by sex (male, female), racial and ethnic group (Black, Hispanic, non-Hispanic White), and age (years), grouped as follows: ages 15 to 19, 20 to 24, 25 to 29, 30 to 34, and 35 to 39 years. To protect the privacy of decedents, the Illinois Department of Public Health does not release data on demographic groups with fewer than 10 firearm deaths per year. Therefore, for each age group, we compared the *NJP* sample to Cook County from the years in which our participants in that age group died. For example, for the group ages 20 to 24 years, participants in the *NJP* sample died between 2000 and 2006; thus, we obtained aggregated counts of firearm death of individuals ages 20 to 24 years in the general population between 2000 and 2006. For the group ages 25 to 29 years, we used data from 2005 to 2011. Following this approach, we used Cook County data from 2011 to 2017 for individuals ages 30 to 34 years and Cook County data from 2016 to 2020 for individuals ages 35 to 39 years. The only exception was for the group ages 15 to 19 years: those in our sample died between 1995 and 2002 but the earliest data available for the general population started in 2000. Therefore, we used aggregated counts of firearm death of individuals ages 15 to 19 years in the general population from 2000 to 2002.

Incarceration

© 2023 Zheng N et al. *JAMA Network Open.*

Incarceration variables are based on data from official records. We obtained intake and exit dates for correctional stays from the Illinois Department of Corrections adult and youth divisions, the Cook County Department of Corrections, and the Clerk of the Court of Cook County (for stays in the CCJTDC). Because it was not feasible to collect records for those in federal prisons, out-of-state prisons, and detention facilities outside of Cook County, dates for stays in these facilities are based on self-report (<3% of stays).

We matched official records to participants using names, known aliases, race and ethnicity, birth dates, and institutional ID numbers. Throughout the course of the study, we routinely updated identifying information each time a participant was contacted or interviewed. We also routinely updated institutional IDs every time a participant was interviewed in a correctional setting or discovered to be incarcerated through multiple mailings sent to participants each year of the study.

Attrition

Through December 2020, we collected data on death for all participants, regardless of whether or not they could be located for interviews. We obtained information on deaths through (1) death certificates from state medical examiners' offices; (2) online news sources; (3) the National Death Index; and (4) reports from participants' families, friends, and acquaintances. For the n = 88 participants who withdrew or refused participation, we censored them on their last interview date. We collected data on nonfatal firearm injury at every follow-up interview up through the 16-year interview. For participants who died (n = 120), withdrew (n = 88), could not be located by the 16-year interview (n = 220), or whose 16-year interview was conducted out of range (n = 13), we considered them censored for nonfatal firearm injury at their most recent interview date.

We conducted follow-up interviews at approximately 3, 5, 6, 8, 12, 14, 15, and 16 years after the baseline interview for the entire sample. Depending on wave, participation ranged from 91% (n = 1659, 3 years after baseline) to 73% (n = 1333, 8 years after baseline). Sixteen years after baseline, we interviewed 82% of participants who were still alive (n = 1388, 76% of the original sample): 860 male; 528 female; 809 Black; 374 Hispanic; 203 Non-Hispanic white; and 2 other race and ethnicity; median age 32 years old. Females were more likely to be retained 16 years after detention compared with males (odds ratio [OR], 1.48; 95% CI, 1.18-1.87). Black participants were more likely to be retained 16 years after detention compared with non-Hispanic whites (OR, 1.89; 95% CI, 1.41-2.53) and Hispanics (OR, 1.66; 95% CI, 1.29-2.12).

STATISTICAL ANALYSES

Poisson Models

We used Poisson regression with a log offset for time at risk to: (1) generate the incidence rates and associated 95% CIs of nonfatal firearm injury, and firearm injury and mortality, and (2) compare rates of firearm injury and mortality by sex and race and ethnicity (self-reported). We estimated incidence rates separately for males and females. As in a prior article,[15] when

estimating sex and racial ethnic differences, we defined time at risk to include only days lived in the community. We excluded days incarcerated from time at risk because incarceration greatly reduces the risk of firearm injury and death; none of our participants were injured or killed by firearms while incarcerated. This approach reduces potential confounding effects of sex and race and ethnicity, because males and racial and ethnic minorities are disproportionately incarcerated.[16]

Some participants reported having been injured by a firearm at more than one interview; others were injured by a firearm and, in a separate incident, killed by a firearm. For these participants, we included the first injury in our analyses of rates of firearm injury and mortality.

© 2023 Zheng N et al. *JAMA Network Open.*

eTable. Unweighted Sample Characteristics at Baseline

Characteristic	Baseline (n= 1829) N	(%)[a]
**Race and Ethnicity**		
Black	1005	(55)
Hispanic	524	(29)
Non-Hispanic White	296	(16)
Other	4	(0)
**Sex**		
Male	1172	(64)
Black	575	(49)
Hispanic	387	(33)
Non-Hispanic White	207	(18)
Other	3	(0)
Female	657	(36)
Black	430	(65)
Hispanic	137	(21)
Non-Hispanic White	89	(14)
Other	1	(0)
**Legal Status at Detention**		
Processed in adult court	275	(15)
Processed in juvenile court	1554	(85)
**Age, years**		
Mean (SD)	14.9	(1.4)
Median	15	
Range	10 - 18	

[a] Percentages may not sum to 100% due to rounding error.

© 2023 Zheng N et al. *JAMA Network Open.*

eFigure. Standardized Firearm Mortality Rates in the Northwestern Juvenile Project (NJP) Compared With the General Population

	NJP Sample		General Population			
	Rate[a]	95% CI	Rate[b]	95% CI	Rate Ratio	95% CI[c]
Total[d]						
15 - 19 y	666	(390, 1137)	95	(82, 107)	**7.0**	**(4.1, 12.2)**
20 - 24 y	499	(259, 960)	183	(170, 195)	**2.7**	**(1.4, 5.3)**
25 - 29 y	337	(150, 758)	118	(108, 129)	**2.9**	**(1.3, 6.4)**
30 - 34 y	115	(32, 405)	119	(108, 131)	1.0	(0.3, 3.4)
35 - 39 y	220	(70, 691)	110	(97, 124)	2.0	(0.6, 6.3)
Male[d]	411	(291, 580)	141	(135, 147)	**2.9**	**(2.1, 4.1)**
15 - 19 y	708	(412, 1219)	102	(88, 115)	**7.0**	**(4.0, 12.2)**
20 - 24 y	537	(278, 1037)	196	(183, 210)	**2.7**	**(1.4, 5.3)**
25 - 29 y	359	(157, 817)	127	(116, 138)	**2.8**	**(1.2, 6.5)**
30 - 34 y	124	(35, 439)	128	(115, 141)	1.0	(0.3, 3.4)
35 - 39 y	236	(75, 744)	118	(104, 133)	2.0	(0.6, 6.3)
Black	456	(308, 676)	168	(161, 175)	**2.7**	**(1.8, 4.0)**
15 - 19 y	758	(401, 1431)	114	(98, 132)	**6.6**	**(3.5, 12.7)**
20 - 24 y	577	(267, 1246)	232	(216, 250)	**2.5**	**(1.1, 5.4)**
25 - 29 y	438	(185, 1034)	152	(139, 167)	**2.9**	**(1.2, 6.8)**
30 - 34 y	138	(33, 572)	154	(139, 170)	0.9	(0.2, 3.7)
35 - 39 y	280	(83, 948)	143	(125, 162)	2.0	(0.6, 6.7)
Non-Hisp White	107	(43, 267)	4	(4, 5)	**24.7**	**(9.7, 62.6)**
15 - 19 y	-	- -	9	(5, 14)	-	- -
20 - 24 y	-	- -	8	(6, 11)	-	- -
25 - 29 y	-	- -	4	(2, 5)	-	- -
30 - 34 y	-	- -	2	(1, 4)	-	- -
35 - 39 y	-	- -	2	(1, 4)	-	- -
Hispanic	286	(180, 453)	30	(27, 33)	**9.6**	**(6.0, 15.3)**
15 - 19 y	663	(357, 1234)	63	(51, 77)	**10.5**	**(5.5, 20.1)**
20 - 24 y	485	(231, 1016)	48	(41, 55)	**10.1**	**(4.8, 21.5)**
25 - 29 y	-	- -	20	(16, 25)	-	- -
30 - 34 y	-	- -	17	(14, 22)	-	- -
35 - 39 y	-	- -	16	(12, 22)	-	- -
Female[d]	49	(23, 103)	7	(5, 8)	**7.4**	**(3.4, 15.9)**
15 - 19 y	126	(47, 341)	6	(3, 9)	**20.7**	**(6.8, 63.0)**
20 - 24 y	-	- -	10	(7, 13)	-	- -
25 - 29 y	-	- -	5	(3, 6)	-	- -
30 - 34 y	-	- -	5	(3, 7)	-	- -
35 - 39 y	-	- -	6	(3, 9)	-	- -
Black	52	(21, 124)	8	(7, 10)	**6.1**	**(2.5, 15.0)**
Non-Hisp White	-	- -	1	(0, 1)	-	- -
Hispanic	-	- -	2	(1, 2)	-	- -

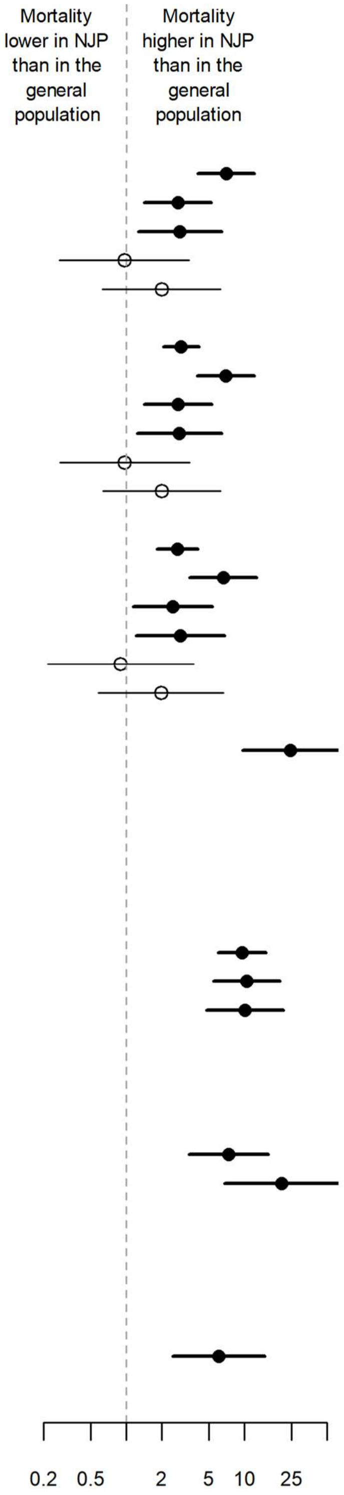

A dash indicates that there were 3 or fewer deaths in the NJP sample.
Bold text, statistically significant difference in mortality rates ($p < 0.05$).
[a] Deaths per 100,000 person-years.
[b] Deaths per 100,000 person-years in Cook County.
[c] Standard error estimated via the delta method.
[d] Rates across racial and ethnic categories are weighted to reflect the racial and ethnic distribution of the Cook County Juvenile Temporary Detention Center.

© 2023 Zheng N et al. *JAMA Network Open.*

eAppendix 1. Literature Table: Studies of Firearm Injury or Mortality in Youth Involved With the Juvenile Justice System Since 1990, United States

Authors, Publication Year	Sample					Design	Assessed Firearm Injury or Death?[1]	Reported Prevalence Rates of Firearm Injury or Mortality?	Reported Incidence Rates of Firearm Injury or Mortality?
	Sample and Location	Sampling Strategy	% Racial/ Ethnic Minority	Sex	Age at Baseline (yrs)				
Teplin et al, 2014[15]	1829 youth detained, Cook County Juvenile Temporary Detention Center, Chicago, IL	Random Sample	63% B; 27% H	55% M; 45% F	10-18	Longitudinal (7-16 years)	Firearm homicide	Yes	No
Ezell & Tanner-Smith, 2009[17]	4866 youth incarcerated, California Youth Authority	Random Sample	38% B; 30% H; 3% other	100% M	Median 20	Review of Death Records (8-18 years after release)	Firearm homicide	Yes	No
Ramchand et al, 2009[18]	449 youth detained, juvenile detention facilities, Los Angeles, CA	Convenience Sample	55% H; 15% B; 14% other	87% M; 13% F	13-17	Longitudinal (7 years)	Firearm injury; Firearm death	Yes	No
Teplin et al, 2005;[19]	1829 youth detained, Cook County Juvenile Temporary Detention Center, Chicago, IL	Random Sample	63% B; 27% H	55% M; 45% F	10-18	Longitudinal (7-16 years)	Firearm homicide	Yes	No
Callahan et al, 1993[20]	89 youth detained, short-term holding facility, King County, WA,	Convenience Sample	43% B; 11% A; 10% other	100% M	15-18	Cross-sectional	Self-inflicted firearm injury	Yes	No

Abbreviations: A, Asian; B, Black; H, Hispanic; F, Female; M, Male

[1] Firearm death includes firearm homicide as well as suicide by firearm, accidental shootings, and legal interventions.

© 2023 Zheng N et al. *JAMA Network Open.*

eAppendix 2. Summary of Nonfatal Firearm Injury Data in the General Population

We could not compare our rates of nonfatal firearm injury with the general population because there are no comparable data. The state level Trauma Registry Database[21] reports firearm injuries only among patients presenting to Level I and II trauma centers in Illinois between 1994 and 2013. This registry excludes victims who are untreated or treated in other settings (e.g., community hospitals, doctor's offices, or emergency care clinics). Therefore, these data substantially underestimate nonfatal firearm injury in the general population. The national level databases, National Electronic Injury Surveillance System (NEISS) and Web-based Injury Statistics Query and Reporting System (WISQARS), cannot provide county-level data. Furthermore, NEISS cannot provide estimates by race and ethnicity due to considerable missing data.[22]

eReferences

1. Pastore A, Maguire K. *Sourcebook of Criminal Justice Statistics - 1999.* Washington, DC2000.
2. US Bureau of the Census. *The Hispanic Population.* Washington, DC.
3. Snyder H, Sickmund M. *Juvenile Offenders and Victims: 1999 National Report.* Washington, DC1999.
4. Snyder HN, Sickmund M. *Juvenile Offenders and Victims: 2006 National Report.* Washington, DC: U.S. Department of Justice, Office of Justice Programs, Office of Juvenile Justice and Delinquency Prevention;2006.
5. Teplin LA, Abram KM, McClelland GM, Dulcan MK, Mericle AA. Psychiatric disorders in youth in juvenile detention. *Arch Gen Psychiatry.* 2002;59(12):1133-1143.
6. Puzzanchera C, Stahl A, Finnegan TA, Tierney N, Snyder HN. *Juvenile Court Statistics 1999.* Pittsburg PA: Office of Juvenile Justice and Delinquency Prevention;2003. NCJ # 201241.
7. General Accounting Office. *Juvenile Justice: Juveniles Processed in Criminal Court and Case Dispositions.* Washington, DC: US General Accounting Office;1995. GAO/GGD-95-170.
8. Griffin P. *Trying and sentencing juveniles as adults: an analysis of state transfer and blended sentencing laws. Technical Assistance to the Juvenile Court: Special Project Bulletin.* Pittsburg, PA: National Center for Juvenile Justice;2003.
9. Snyder HN, Sickmund M, Poe-Yamagata E. *Juvenile Transfers to Criminal Court in the 1990's: Lessons Learned From Four Studies.* Washington, DC: US Department of Justice, Office of Juvenile Justice and Delinquency Prevention;2000.
10. Salekin RT, Yff R, Neumann CS, Leistico A-MR, Zalot AA. Juvenile transfer to adult courts: a look at the prototypes for dangerousness sophistication-maturity and amenability to treatment through a legal lens. *Psychology, Public Policy, and Law.* 2002;8(4):373-410.
11. Austin J, Johnson KD, Gregoriou M. *Juveniles in Adult Prisons and Jails: A National Assessment.* Washington, DC: Office of Juvenile Justice and Delinquency Prevention;2000.
12. Washburn JJ, Teplin LA, Voss LS, Simon CD, Abram KM, McClelland GM. Psychiatric disorders among detained youths: a comparison of youths processed in juvenile court and adult criminal court. *Psychiatric Services.* 2008;59(9):965-973.
13. Federal Policy for the Protection of Human Subjects: Notices and Rules: Part 2. *Federal Register.* 1991;56(117):28002-28032.
14. Fisher CB. Integrating science and ethics in research with high-risk children and youth. *Social Policy Report.* 1993;7(4):1-27.
15. Teplin LA, Jakubowski JA, Abram KM, Olson ND, Stokes ML, Welty LJ. Firearm homicide and other causes of death in delinquents: a 16-year prospective study. *Pediatrics.* 2014;134(1):63-73.
16. Willaim J Sabol, Thaddeus L Johnson, Caccavale A. Trends in correctional control by race and sex. 2019. Accessed July 23, 2021. https://cdn.ymaws.com/counciloncj.org/resource/collection/4683B90A-08CF-493F-89ED-A0D7C4BF7551/Trends_in_Correctional_Control_-_FINAL.pdf.
17. Ezell ME, Tanner-Smith EE. Examining the role of lifestyle and criminal history variables on the risk of homicide victimization. *Homicide Studies.* 2009;13(2):144-173.
18. Ramchand R, Morral AR, Becker K. Seven-year life outcomes of adolescent offenders in Los Angeles. *American Journal of Public Health.* 2009;99(5):863-870.
19. Teplin LA, McClelland GM, Abram KM, Mileusnic D. Early violent death among delinquent youth: a prospective longitudinal study. *Pediatrics.* 2005;115(6):1586-1593.
20. Callahan CM, Rivara FP, Farrow JA. Youth in detention and handguns. *J Adolesc Health.* 1993;14:350-355.
21. Trauma Registry Database. Accessed Mar, 2nd, 2021. http://app.idph.state.il.us/emsrpt/trauma.asp.

© 2023 Zheng N et al. *JAMA Network Open.*

22. Fowler KA, Dahlberg LL, Haileyesus T, Annest JL. Firearm injuries in the United States. *Preventive Medicine.* 2015;79:5-14.

© 2023 Zheng N et al. *JAMA Network Open.*
