# Supplementary material for: Nonfatal Firearm Injury and Firearm Mortality in High-risk Youths and Young Adults 25 Years After Detention
Source: JAMA Netw Open. 2023 Apr 21;6(4):e238902. doi: 10.1001/jamanetworkopen.2023.8902 (PMC10122168; doi:10.1001/jamanetworkopen.2023.8902)
Supplement: Supplement 2. — Data Sharing Statement [file jamanetwopen-e238902-s002.pdf]

Data Sharing Statement

Zheng. Nonfatal Firearm Injury and Firearm Mortality in High-Risk Youths and Young Adults 25 Years After Detention. *JAMA Netw Open*. Published April 21, 2023.
doi:10.1001/jamanetworkopen.2023.8902

Data
Data available: Yes
Data types: Deidentified participant data, Data dictionary
How to access data: Partial study data available at ICPSR: https://www.icpsr.umich.edu/web/NACJD/search/studies?start=0&ARCHIVE=NACJD&PUBLISH_STATUS=PUBLISHED&sort=score%20desc%2CTITLE_SORT%20asc&rows=50&q=northwestern%20juvenile%20project. Deidentified study data used in the manuscript will be deposited to ICPSR upon publication.
When available: With publication

Supporting Documents
Document types: None

Additional Information
Who can access the data: Researchers whose proposed use of the data has been approved
Types of analyses: For research purposes approved by researcher's institutional IRB
Mechanisms of data availability: After approval of a proposal by ICPSR
